# The C-Terminus of ClpC1 of *Mycobacterium tuberculosis* Is Crucial for Its Oligomerization and Function

**DOI:** 10.1371/journal.pone.0051261

**Published:** 2012-12-20

**Authors:** Divya Bajaj, Janendra K. Batra

**Affiliations:** Immunochemistry Laboratory, National Institute of Immunology, Aruna Asaf Ali Marg, New Delhi, India; University of Delhi, India

## Abstract

*Mycobacterium tuberculosis* ClpC1 is a member of the Hsp100/Clp AAA+ family of ATPases. The primary sequence of ClpC1 contains two N-terminal domains and two nucleotide binding domains (NBD). The second NBD has a long C-terminal sub-domain containing several motifs important for substrate interaction. Generally, ClpC proteins are highly conserved, however presence of C-terminal domains of variable lengths is a remarkable difference in ClpC from different species. In this study, we constructed deletion mutants at the C-terminus of *M. tuberculosis* ClpC1 to determine its role in the structure and function of the protein. In addition, a deletion mutant having the two conserved N-terminal domains deleted was also constructed to investigate the role of these domains in *M. tuberculosis* ClpC1 function. The N-terminal domains were found to be dispensable for the formation of oligomeric structure, and ATPase and chaperone activities. However, deletions beyond a specific region in the C-terminus of the ClpC1 resulted in oligomerization defects and loss of chaperonic activity of the protein without affecting its ATPase activity. The truncated mutants, defective in oligomerization were also found to have lost the chaperonic activity, showing the formation of oligomer to be required for the chaperonic activity of *M. tuberculosis* ClpC1. The current study has identified a region in the C-terminus of *M. tuberculosis* ClpC1 which is essential for its oligomerization and in turn its function.

## Introduction

Molecular chaperones mediate correct folding, assembly, transport as well as disaggregation of proteins inside the cell during growth in normal as well as stressful conditions. Many molecular chaperones are expressed at higher level during biological stress and have been classified under heat shock proteins (Hsps) [1]–[3]. The heat shock proteins are further grouped into five major families namely, Hsp100, Hsp90, Hsp70, Hsp60 and small heat shock protein (sHsps), based on their molecular masses [4]. The Hsp100 family consists of a group of AAA+ (ATPases associated with cellular activities) family of ATP-dependent chaperones that transfers misfolded proteins for degradation into the proteolytic chamber of an associated protease [5], [6]. These proteases also known as caseinolytic proteases or Clps are hetero-oligomers of an ATPase component belonging to the Hsp100 family and a protease component known as ClpP. Clps play an essential role in protein turnover [7], [8]. Clps belonging to Hsp100 family have either one or two copies of the conserved ATPase (AAA+) core domain. The Hsp100 members containing two ATPase domains have been grouped under class I and include ClpA-E and L, whereas class II members that include ClpX and ClpY contain one ATPase domain [3], [7]. The ATPase domain consists of a Walker A and Walker B motif [9]. The Hsp100 proteins, ClpX and ClpY form a hexameric ring that attaches with a barrel shaped protease, ClpP or ClpQ which is responsible for the substrate hydrolysis [7].

Clp proteins have been shown to play a role in the virulence of many pathogenic bacteria. In *Listeria monocytogenes*, ClpC has been shown to be important for its virulence and survival in macrophages, and in *Bacillus subtilis* it controls the competence gene expression and survival under stressful conditions [10]–[12]. ClpC regulates transcription of the major virulence factor in *Staphylococcus aureus* [13]. In *Mycobacterium tuberculosis*, ClpC1 was shown to be up regulated upon re-exposure to favorable conditions after hypoxia along with ClgR, a Clp protease gene regulator [14]. ClpP mutation was shown to significantly attenuate the virulence of *Streptococcus pneumoniae* in a murine intraperitoneal infection model [15]. In *Staphylococcus aureus*, ClpP deletion caused complete down-regulation of heat shock regulon and partial depression of genes involved in oxidative stress indicating a strong impact of ClpP proteolytic activity on virulence and stess response [16]. The partial disruption of Hsp regulation in *M. tuberculosis* impaired the ability of the bacteria to establish a chronic infection as compared to the wild type [17].

The *M. tuberculosis* H37Rv ClpC1 is an ATP-dependent molecular chaperone which belongs to the class I of Hsp100 family of AAA+ proteins [18]. Earlier, we have shown *M. tuberculosis* ClpC1 to manifest chaperonic activity *in vitro* in the absence of any adaptor protein [18]. *M. tuberculosis* ClpC1 has been shown to interact with RseA, an anti sigma factor, and ClpC1P2 complex has been shown to proteolytically cleave RseA *in vitro* [19]. The knockdown of ClpC1 in *M. smegmatis* and *M. tuberculosis* resulted in an inhibition of RseA degradation [19]. Recently, ClpC1 of *M. tuberculosis* has been shown to associate with ClpP1P2 complex to degrade the model substrate casein in the presence of some activators *in vitro* [20].

**Figure 1:**
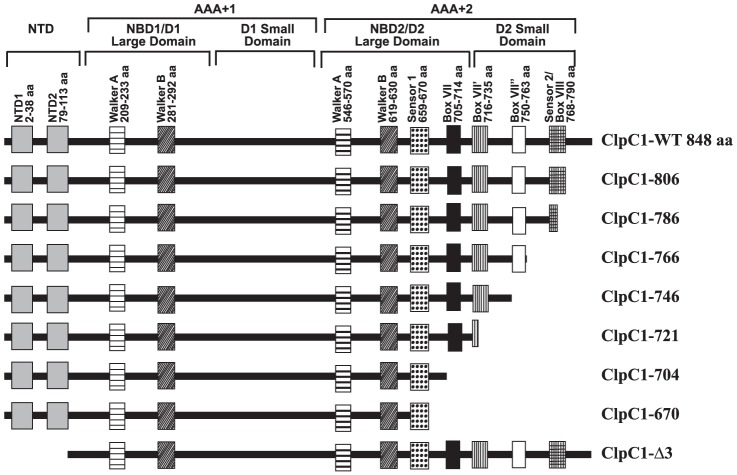
Diagrammatic representation of ClpC1-WT and its mutants used in the study. Various conserved regions within NTD, D1 and D2 domains namely N-terminal repeats, Walker A domain, Walker B domain, Sensor I, Box VII, Box VII′, Box VII″ and Box VIII are indicated. ClpC1-WT and deletion mutants ClpC1–806, ClpC1–786, ClpC1–766, ClpC1–746, ClpC1–721, ClpC1–704, ClpC1–670 and ClpC1-Δ3 are also represented.

ClpC proteins are most highly conserved subgroups within the Clp family. ClpC1 of *M. tuberculosis* consists of two N-terminal domains (NTD) and two AAA+ domains (Figure 1). The AAA+2 domain has a long carboxy-terminal sub-domain proposed to interact with protein substrates, which has been also termed as the substrate sensor and discrimination (SSD) domain [21]. A common feature of ClpC proteins is the presence of sensor residues toward the C-terminus, which function to sense the presence of a *γ*-phosphate in the nucleotide and to catalyze nucleotide hydrolysis. The carboxy-terminus of Clp proteins have been shown by Neuwald *et al*. [22] to have several conserved motifs, which include Sensor 1, Box VII, Box VII′, Box VII″ and Sensor 2/Box VIII (Figure 1). The sensor 1 motif has a conserved Asn or Thr residue that could hydrogen bond with phosphate of the nucleotide [23]. The Box VII has a conserved Arg that interacts with the nucleotide phosphate group. The Box VIII motif also has a conserved Arg residue which is involved in substrate interaction [24].

**Figure 2:**
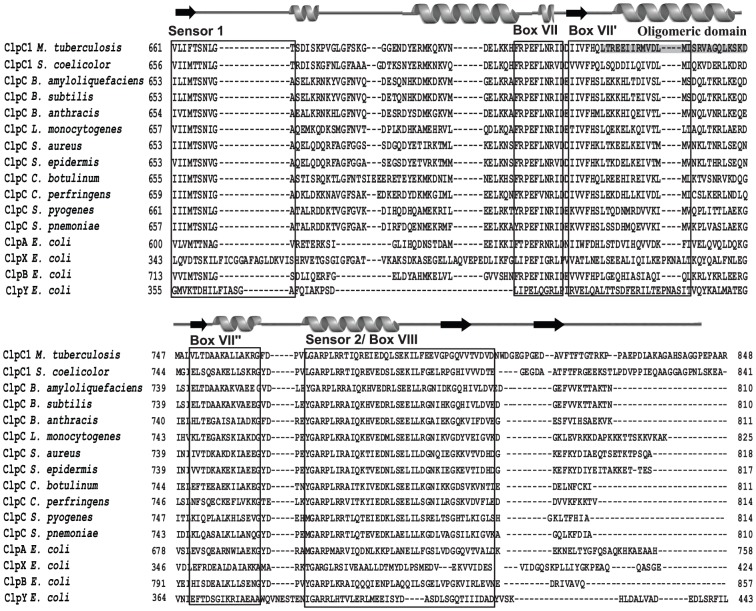
Sequence alignment of C-terminus of *M. tuberculosis* ClpC1 with other Clp proteins. Structure-based multiple sequence alignment of *M. tuberculosis* ClpC1 was done using PROMALS3D software with the C-terminal amino acid sequences of Clps of other bacteria. The various conserved regions are boxed and labeled. The observed secondary structures are shown on top. The α-helices are shown by helices and β-sheets by arrows. The region identified by this study responsible for oligomerization in *M tuberculosis* ClpC1 is highlighted.

Although, generally ClpC proteins are highly conserved subgroup within the Clp family, there are a few remarkable differences in different species. One such difference is the presence of C-terminal domain of variable lengths. As compared to Clps of other bacteria, the *M. tuberculosis* ClpC1 has a long C-terminal extension beyond the conserved sensor 2/Box VIII (Figure 2). In this study, we report a structure-function analysis aimed at identifying the role of C-terminus of *M. tuberculosis* ClpC1 in its functional activity. The study shows that the C-terminus plays a crucial role in ClpC1 function as it is involved in the oligomerization of the protein which is a pre-requisite for its activity. A detailed understanding of ClpC structure-function relationship has implications in exploring ClpC as a drug target in tuberculosis.

## Experimental Procedures

### Construction of *M. tuberculosis* ClpC1 mutants

The ClpC1 deletion mutants were constructed using previously cloned ClpC1 in pVex11 as template [18]. The C-terminal deletion mutants, ClpC1–806 (Δ807–848 aa), ClpC1–786 (Δ787–848 aa), ClpC1–766 (Δ767–848 aa), ClpC1–746 (Δ747–848 aa), ClpC1–721 (Δ722–848 aa), ClpC1–704 (Δ705–848 aa) and ClpC1–670 (Δ671–848 aa) were constructed by PCR using a common 5′ primer and reverse primers with termination codon at the desired location (Table 1). For the N-terminal truncated mutant, ClpC1-Δ3 a forward primer was designed with a start codon before the 114^th^ amino acid (Table 1). The PCR amplified DNAs were digested with *Nde*I and *Hind*III and cloned into a T7 promoter-based expression vector, pVex11 digested with the same enzymes [18]. The sequences of the constructs were confirmed by DNA sequencing.

**Table 1:** Primers used for the amplification of truncated mutants of *M. tuberculosis* ClpC1.

Constructs	Direction of the primer	Primer Sequence
ClpC1–806	reverse	5′ ATAATCTACAAGCTTCTAGTTGTCCACGTCCAG 3′
ClpC1–786	reverse	5′ ATAATCTACAAGCTTCTAGGTGCCCAGATTGGA 3′
ClpC1–766	reverse	5′ ATAATCTACAAGCTTCTAGTCCTTGCTCTTGAG 3′
ClpC1–746	reverse	5′ ATAATCTACAAGCTTCTACGGGTCGAAGCCACG 3′
ClpC1–721	reverse	5′ ATAATCTACAAGCTTCTACTGGTGGAAGACGAT 3′
ClpC1–704	reverse	5′ ATAATCTACAAGCTTCTAGTGTTTCTTCAGCTC 3′
ClpC1–670	reverse	5′ ATAATCTACAAGCTTCTACGAGAGCTGATCTTC 3′
ClpC1-Δ3	forward	5′ GTAGATACATATGCGAGAGGGTGAAGGCGTG 3′
All C-term constructs*	forward	5′ ACTCACTATAGGGAGACCAC 3′

The restriction endonuclease recognition sites are underlined.

* The forward primer, annealing to pVex11 vector upstream of the T7 promoter used for the amplification of all C-terminal deletion mutants.

### Expression and purification of recombinant *M. tuberculosis* ClpC1 and its mutants

ClpC1-WT and all ClpC1 mutants were expressed in *E. coli* BL21 cells in superbroth at 30°C and induced with 1 mm isopropyl thio-β-D-galactopyranoside for 3 hours as described earlier [18] Cells were lysed in a lysis buffer containing 50 mM Tris-HCl, pH 7.8, 200 mM KCl, 5 mM dithiothreitol, 10% (w/v) sucrose, 10% glycerol, 30 mM Spermidine–HCl and 1 mg/mL lysozyme by incubation on ice for 45 min. To ensure complete lysis, the concentration of KCl was increased to 1 M and the mixture was further incubated at 42°C for 5 min. The lysate was centrifuged at 40,000 g for 30 min at 4°C. The supernatant was further centrifuged at 1,00,000 g for 1 h at 4°C. All mutants, like the wild type protein, were localised in the soluble cytosolic fraction. The cytosolic fraction was dialysed against buffer A, composed of 50 mM Tris-HCl, pH 7.6, 100 mM KCl, 5 mM dithiothreitol, 10% (v/v) glycerol and 0.01% Triton X-100. The dialysed supernatant was loaded onto a Q-Sepharose column equilibrated with the same buffer. The bound proteins were eluted with a salt gradient of 0.1 to 1.5 M KCl in buffer A using a GE-AKTA-Basic chromatography system. The ClpC1 proteins were further fractionated by 40% ammonium sulphate precipitation and purified to near homogeneity using a Superdex-200 (GE Healthcare, Piscataway, NJ, USA) column equilibrated with buffer A.

### ATPase activity assay

For a standard ATPase assay, 4 µg Clp protein was incubated in a 50 µl reaction mixture containing buffer A, 10 mM ATP containing [^32^γP] ATP and 10 mM MgCl_2_ at 37°C for 30 min. The reaction was stopped by adding 50 µl of chilled activated charcoal (100 mg/ml in 1 M HCl). The mixture was incubated on ice for 5 min with intermittent shaking, and centrifuged at 4°C at 16,000 g for 15 min. Radioactivity in the supernatant was measured using a liquid scintillation counter, and the concentration of released Pi was calculated using the specific activity of the radioactive substrate. The standard errors (SE) of means were calculated using the Sigma plot software.

### Prevention of aggregation of luciferase

The heat induced aggregation of luciferase was monitored in a buffer containing 50 mM HEPES-KOH, pH 7.6, 10% (v/v) glycerol, 5 mM dithiothreitol, 10 mM MgCl_2_ and 25 mM KCl in a Varian Cary Eclipse fluorescence spectrophotometer equipped with a temperature controller at 43°C at 320 nm. Various concentrations of ClpC1 and its mutants, with or without 5 mM ATP, were added in the reaction and their effect on the aggregation was assessed.

### Reactivation of heat aggregated luciferase

Luciferase was denatured by incubating at 43°C for 15 min. To measure reactivation of luciferase, in a 50 µl reaction, 5 nM heat-denatured luciferase was incubated at 25°C with various concentrations of ClpC1 and its mutants in the presence of 10 mM ATP and 10 mM MgCl_2_ for 45 min. The refolding of denatured luciferase by Clp proteins was analyzed by assaying the luciferase activity. The luciferase activity was measured in a buffer containing 20 mM Tris-HCl pH 7.8, 1.1 mM MgCl_2_, 2.7 mM MgSO_4_, 0.1 mM EDTA, 33 mM DTT, 530 µM ATP pH 7.8 and 270 µM Coenzyme A. The substrate Luciferin was added to a final concentration of 470 µM in a Berthold MicroLumat luminometer and luminescence was recorded. The activity of luciferase which was not heat inactivated was considered as 100%. The activity of heat inactivated luciferase, incubated without ClpC1 protein was considered as the background. The standard errors (SE) of means were calculated using the Sigma plot software.

### CD spectral analysis of proteins

For CD spectral analysis, spectra of 4 µM solution of proteins in buffer A were recorded in the far-UV range (200–250 nm) at 30°C using a JASCO J-710 spectropolarimeter. A cell with a 1 cm optical path was used to record the spectra at a scan speed of 200 nm min^−1^ with a sensitivity of 50 mdeg and a response time of 1 s. The sample compartment was purged with nitrogen, and spectra were averaged over 10 scans. The results are presented as mean residue ellipticity (MRE).

### Gel-filtration chromatography

To analyze the oligomeric status of proteins, they were applied onto a 1×30 cm Superdex-200 column equilibrated with buffer A. The column was run at a constant flow rate of 0.4 ml min^−1^using a GE-AKTA-Prime chromatography system. If chromatography was performed in the presence of ATP the proteins were incubated with 15 mM ATP and 10 mM MgCl_2_ for 30 min at 25°C before loading onto the column, and buffer A containing 15 mM ATP and 10 mM MgCl_2_ was used to run the column.

## Results

### Sequence analysis of ClpC1 of *M. tuberculosis*

ClpC1 of *M. tuberculosis* is a 848 amino acid long protein containing two NTDs, two NBDs that have walker A and Walker B motif and a C-terminal domain (Figure 1). The N-terminal domains and the ATPase domains are generally conserved among different organisms. After the two N-terminal domains, the two ATPase domains are present in D1 and D2 domains respectively (Figure 1). These domains are further divided as D1 large domain (residues 154–350), D1 small domain (residues 351–464), D2 large domain (residues 465–722) and D2 small domain (residues 723–848) (Figure 1) [7], [18]. After the D2 walker B motif beyond amino acid 625, there is a long C-terminal domain. The C-terminal domain contains five conserved motifs, namely Sensor1, Box VII, Box VII′, Box VII″ and Sensor 2/Box VIII (Figure 1). Sequence comparison of *M. tuberculosis* ClpC1 revealed a significant sequence homology at the C-terminus with ClpC, ClpA and ClpB of other bacteria (Figure 2). The sequence is conserved till a motif, DVDN at 802 amino acid, however beyond the DVDN motif there is a 42 amino acid tail which is shorter or absent in ClpC, ClpA and ClpB of other bacteria (Figure 2). In this study, we have investigated the role of C-terminus of ClpC1 of *M. tuberculosis* in its function.

### Construction, expression and characterization of *M. tuberculosis* ClpC1 mutants

We prepared seven ClpC1 mutants having deletions at C-terminus to study the role of conserved as well as unconserved regions (Figure 1). ClpC1–806 has amino acid 807 to 848 deleted from C-terminus. Similarly ClpC1–786 (Δ787–848), ClpC1–766 (Δ767–848), ClpC1–746 (Δ747–848), ClpC1–721 (Δ722–848), ClpC1–704 (Δ705–848) and ClpC1–670 (Δ671–848) have deletions at the C-terminus as shown in the parenthesis.

In addition to the C-terminal deletion mutants, another mutant, ClpC1-Δ3 having the two N-terminal domains deleted was also constructed. The ClpC1-Δ3 mutant starts from amino acid 114 of ClpC1. All the mutants were expressed in *E. coli* BL21 cells as reported earlier for the wild type ClpC1 [18].

**Figure 3:**
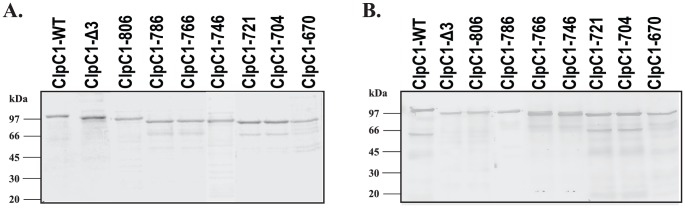
SDS-PAGE and Western blots of purified ClpC1-WT and its deletion mutants. **A.** Purified proteins were analyzed by a 10% SDS-PAGE, **B.** Immunoblot analysis was performed using polyclonal anti-ClpC1 antibody raised in rabbit.

The proteins were found to be localised in the cytosol from where they were purified to near homogeneity by a combination of ammonium sulphate fractionation, anion exchange and gel filtration chromatography, as described earlier [18]. Figure 3A shows the purified proteins as analyzed by SDS-PAGE. All the mutant proteins reacted as well as the ClpC1-WT on a Western blot with an anti-ClpC1 polyclonal antibody (Figure 3B).

**Figure 4:**
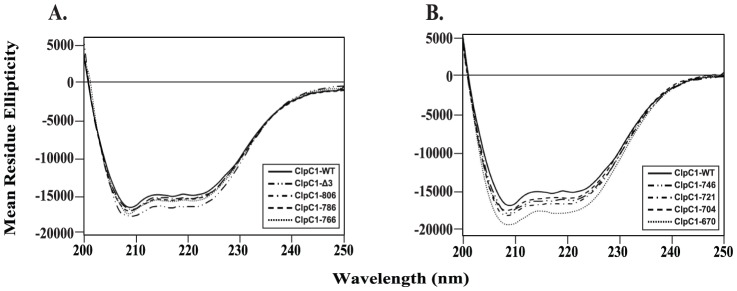
CD-spectral analysis of ClpC1-WT and its deletion mutants. The spectra are presented as mean residue ellipticity, expressed in degree.cm^2^.dmol^−1^. Panel **A** shows spectra of ClpC1-WT, ClpC1-Δ3, ClpC1–806, ClpC1–786 and ClpC1–766, whereas panel **B** shows that of ClpC1-WT, ClpC1–746, ClpC1–721, ClpC1–704 and ClpC1–670.

The effect of deletions on overall ClpC1 structure was investigated by CD spectral analysis of the purified proteins in the far-UV region. ClpC1-WT showed a typical α+β conformation with minima at 208 nm and 224 nm (Figure 4). The CD spectra of ClpC1-WT and its deletion mutants were found to be very similar indicating that the deletions did not affect the overall conformation of the protein (Figure 4).

*M. tuberculosis* ClpC1 has been earlier shown to have an inherent ATPase activity [18]. The deletion mutants were analyzed for their inherent ATPase activity and compared with that of ClpC1-WT (Table 2). The specific activity of ClpC1-WT was found to be 327 units/mg of protein. All the mutants, except ClpC1–786 and ClpC1–746, showed ATPase activity similar to that of the wild type protein (Table 2). The ClpC1–786 and ClpC1–746 mutants had about 2-fold increased ATPase activity (Table 2).

**Table 2:** ATPase activity of *M. tuberculosis* ClpC1 and its mutants.

Clp Protein	ATPase activity (nmol Pi released/mg protein/min) (Mean ± SE)	ATPase activity (%)
ClpC1	327±69	100
ClpC1–806	413±67	126
ClpC1–786	907±78	277
ClpC1–766	345±85	105
ClpC1–746	618±28	189
ClpC1–721	429±11	131
ClpC1–704	471±24	144
ClpC1–670	397±70	121
ClpC1-Δ3	430±82	131

Data represent mean ± SE of three independent experiments.

### Oligomeric status of ClpC1 protein*s*

**Figure 5:**
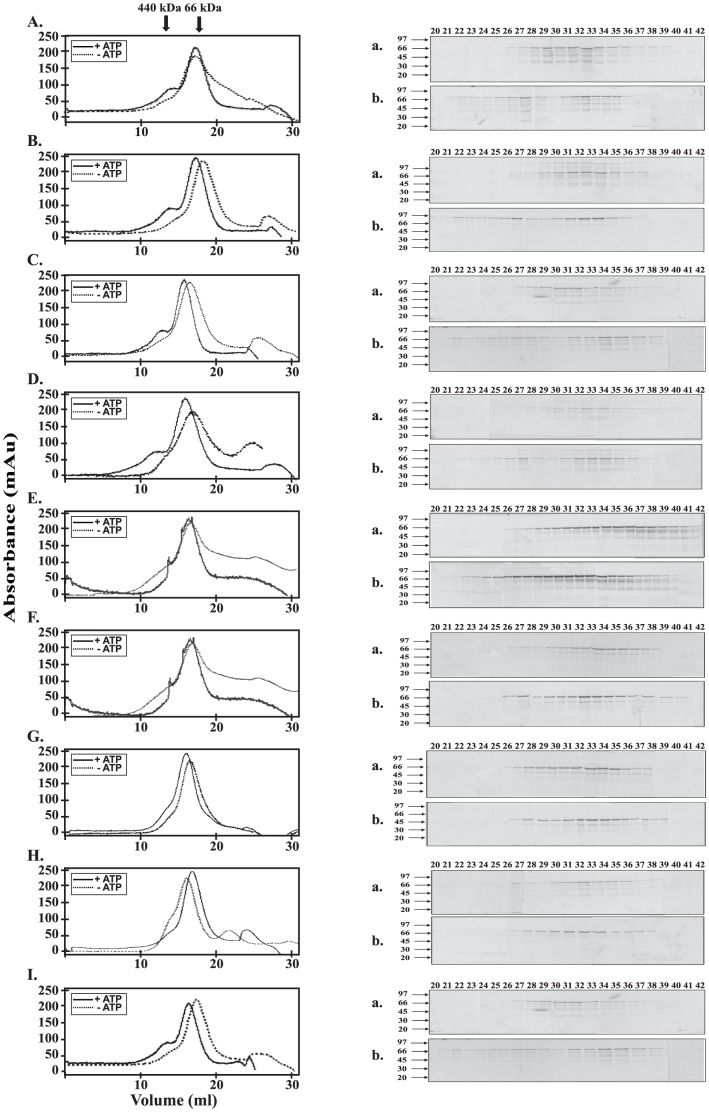
Determination of the oligomeric status of *M. tuberculosis* ClpC1-WT and its deletion mutants. The proteins were run on a 30 cm Superdex 200 column. The elution profile of ClpC1-WT (**A**), ClpC1–806 (**B**), ClpC1–786 (**C**), ClpC1–766 (**D**), ClpC1–746 (**E**), ClpC1–721 (**F**), ClpC1–704 (**G**), ClpC1–670 (**H**), and ClpC1-Δ3 (**I**) are shown. Proteins were analysed in the absence, and in the presence of 15 mM ATP and 10 mM MgCl_2_. The positions of the standards, ferritin (440 kDa) and BSA (66 kDa) are marked by the arrows above. The fractions from the column were analyzed by SDS-PAGE; with ATP (**a**), and without ATP (**b**). Each fraction was 0.5 ml in volume.

Earlier, we have shown that in the absence of ATP *M. tuberculosis* ClpC1 remains predominantly in a monomeric form, however in the presence of ATP a large fraction is shifted towards the hexameric form [18]. In the current study, the oligomerization properties of ClpC1 mutants were compared with that of ClpC1-WT by size exclusion chromatography in the presence and absence of ATP and magnesium chloride. As shown in Figure 5A, ClpC1 showed a peak corresponding to the monomer at 14 ml in the absence of ATP, however in the presence of ATP there was a significant fraction in the hexameric form which appeared as a earlier peak in the chromatogram. When mutants were similarly analyzed for their oligomeric status by size exclusion chromatography in the presence or absence of ATP, ClpC1–806 (Figure 5B), ClpC1–786 (Figure 5C), ClpC1–766 (Figure 5D) and ClpC1–746 (Figure 5E) showed profiles similar to that of ClpC1-WT indicating formation of oligomer in the presence of ATP. However, mutants ClpC1–721 (Figure 5F), ClpC1–704 (Figure 5G) and ClpC1–670 (Figure 5H) did not show the additional peak corresponding to the hexamer in the presence of ATP. The protein fractions obtained from the columns were analyzed by SDS-PAGE and the presence of desired protein was confirmed in the gel filtration column chromatogram peaks. ClpC1-Δ3 with deletion of both the N-terminal domains showed a pattern similar to that of ClpC1-WT (Figure 5I).

### Prevention of luciferase aggregation by ClpC1 and its mutants

**Figure 6:**
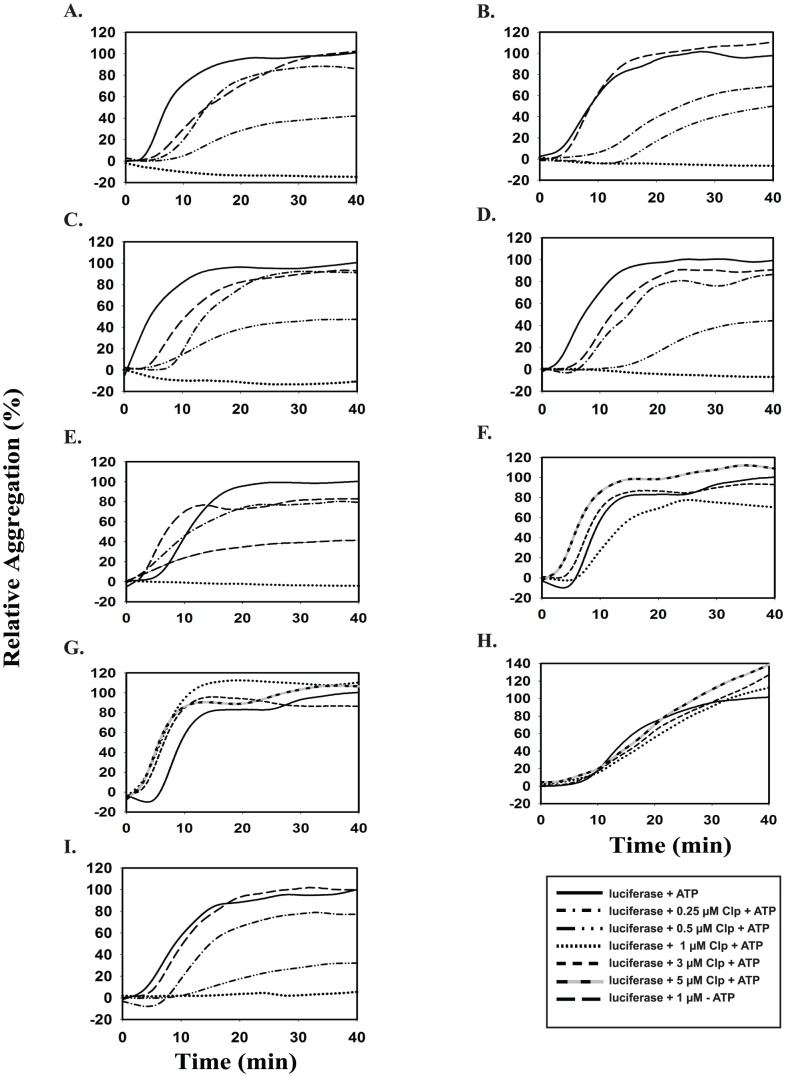
Prevention of luciferase aggregation by *M. tuberculosis* ClpC1-WT and its deletion mutants. Luciferase aggregation was monitored in a buffer with or without Clp proteins at 43°C by following turbidity in a spectrofluorometer at 320 nm. Various panels show data for different proteins. **A.** ClpC1-WT; **B.** ClpC1–806; **C.** ClpC1–786; **D.** ClpC1–766; **E.** ClpC1–746; and **I.** ClpC1-Δ3.

ClpC1 is known to prevent aggregation of luciferase upon heating in an ATP-dependent manner [18]. ClpC1 prevented luciferase aggregation in a dose dependent manner, and in the presence of 1 µM protein almost 100% prevention of luciferase aggregation was observed (Figure 6A). ClpC1 without ATP had no effect on the heat-induced aggregation of luciferase, indicating that ATPase activity of ClpC1 is required for its chaperonic activity (Figure 6A). ClpC1–806 (Figure 6B), ClpC1–786 (Figure 6C), ClpC1–766 (Figure 6D) and ClpC1–746 (Figure 6E) showed a dose dependent prevention of luciferase aggregation activity like the ClpC1-WT, and almost 100% inhibition of aggregation was observed with 1 µM protein. However, ClpC1–721 (Figure 6F), ClpC1–704 (Figure 6G) and ClpC1–670 (Figure 6H) did not show any inhibition of luciferase aggregation with 1 µM protein. Increasing the concentrations of these ClpC1 mutants further upto 5 µM also did not prevent the luciferase aggregation (Figure 6F, G and H). The N-terminal deletion mutant ClpC1-Δ3 had perfectly good prevention of luciferase aggregation activity (Figure 6I).

### Reactivation of heat inactivated luciferase by ClpC1 mutants

Luciferase lost more than 90% of its catalytic activity upon heating at 43°C. Earlier, it was shown that when heat inactivated luciferase is incubated with ClpC1 it is partially reactivated [18]. In this study, ClpC1 lead to about 30% recovery in the activity of heat inactivated luciferase (Table 3). Like ClpC1-WT, ClpC1-Δ3, ClpC1–806, ClpC1–786, ClpC1–766 and ClpC1–746 also showed about 30% recovery of heat inactivated luciferase enzyme activity (Table 3). However, the ClpC1–721, ClpC1–704 and ClpC1–670 mutants showed only about 9%, 7% and 12% reactivation respectively with 2 µM protein (Table 3).

**Table 3:** Reactivation of denatured luciferase by *M. tuberculosis* ClpC1 and its mutants.

Protein	Protein, µM	Luciferase activity (%)
None	-	100 (Unheated)
ClpC1	0.5	13.5±2.5
	1	30.1±5.6
	2	32.4±6.0
ClpC1–806	0.5	12.2±2.2
	1	31.0±5.9
	2	33.5±6.4
ClpC1–786	0.5	12.6±2.5
	1	28.6±5.6
	2	30.2±5.7
ClpC1–766	0.5	12.9±2.5
	1	33.3±6.3
	2	34.3±6.6
ClpC1–746	0.5	11.7±2.1
	1	28.1±5.4
	2	29.0±5.4
ClpC1–721	0.5	0.8±0.9
	1	10.8±2.1
	2	8.8±1.6
ClpC1–704	0.5	1.2±0.2
	1	8.8±1.6
	2	6.8±1.3
ClpC1–670	0.5	0.8±0.2
	1	8.2±1.6
	2	11.6±2.1
ClpC1-Δ3	0.5	13.0±2.4
	1	26.1±4.8
	2	24.7±4.7

Luciferase, 5 nM was heated at 43°C for 15 min with or without the indicated protein. The activity of unheated luciferase was considered as 100% and the activity of heated luciferase, incubated without any protein was considered as the background. Data represent mean ± SE of three independent experiments.

## Discussion

Caseinolytic proteases are ubiquitously present in bacteria, though their distribution varies. Clp proteins have been shown to have a role in the pathogenesis and survival in many pathogenic bacteria. In *Enterococcus faecalis*, ClpB is linked to virulence [25], whereas ClpC has been associated with virulence in *Bacillus subtilis* and *Listeria monocytogenes* [10], [11]. *M. tuberculosis* contains two protease components ClpP1 and ClpP2, and two ATPase components ClpC1 and ClpX of the caseinolytic protease family. In *M. tuberculosis*, ClpP1-ClpP2 knockdown resulted in reduced growth and reduced virulence inside macrophages [26]. Also, in *M. smegmatis*, ClpP1 and ClpP2 were shown to be essential for growth as depletion of either one of them resulted in rapid bacterial death [27]. In *M. tuberculosis* and many Gram positive bacteria ClpC is present as an ortholog of ClpA. It has been seen that interference with the functioning of ClpC1 of *M. tuberculosis* is bactericidal in culture as well as inside the macrophage [28]. Since the Clp proteins in *M. tuberculosis* appear to be essential for survival of the pathogen, a better understanding of their function would help in exploring them as drug targets.

We have earlier shown that ClpC1 self associates to form oligomers in the presence of ATP, and contains inherent ATPase and chaperonic activity [18]. In this study, we have investigated the role of C-terminus of ClpC1 of *M. tuberculosis* in its function. With the help of seven C-terminal deletion mutants of *M. tuberculosis* ClpC1, we demonstrate that extension beyond DVDN motif after amino acid 802 is not required for the functional activity of ClpC1. However, deletion beyond amino acid 746 is detrimental for the chaperonic activity and the oligomerization of the protein. The ATPase activity was not reduced by any of the deletions, on the contrary two mutants, ClpC1–786 and ClpC1–746 had about 2-fold increased ATPase activity. It appears that the deletions in these mutants result in a change in the quaternary structure of the protein, facilitating access of nucleotide to its binding sites. In addition, the study also shows that the deletion of the two N-terminal domains does not affect the *in vitro* chaperonic activity of *M. tuberculosis* ClpC1.

ClpC1 has five conserved motifs, Sensor1, Box VII, Box VII′, Box VII″ and Sensor 2/Box VIII at its C-terminus (Figure 1). Deletion at the C-terminus in ClpC1 upto 746 amino acid was tolerated, however a further deletion upto 721 amino acid resulted in the loss of oligomerization associated with loss of chaperonic activity. The 720–747 amino acid region falls in the small D2 domain of ClpC1 lacking Sensor 2/Box VIII, box VII″ and part of box VII′. The region between box VII to sensor 2 is predicted to form a knob like projection, and in a hexameric ring in Clp proteins these knobs are linked to each other [22]. Our study demonstrates that the region between amino acid 720–747 present in the small D2 domain at the C-terminus is responsible for the oligomerization of *M. tuberculosis* ClpC1 (Figure 1).

In a hexameric ring in AAA+ proteins, the C-terminal domain known as SSD domain contacts its own as well as adjacent subunit's ATPase domain, and is involved in substrate interaction [24]. In ClpB of *E. coli*, the small D2 domain present at the C-terminus of second ATPase domain was found to be required for the oligomerization of the protein [24]. The deletion at the C-terminal domain in ClpB has been shown to inhibit self association, which leads to a decreased ATPase and chaperonic activity [29]. The X-ray crystallographic structure of ClpB revealed that the small D2 domain is responsible for oligomerization as it interacts with the NBD2 domain of the adjacent subunit in a hexamer [30]. In HslU (ClpY), another Hsp100 family protein the C-terminal domain is shown to be involved in the hexamerization of the protein as it interacts with its own core ATPase domain and the adjacent ATPase domain [31]. It has been shown that isolated C-terminal fragments of ClpA, ClpB, ClpX, and ClpY selectively interact with several proteins [32]. It was postulated that the C-terminal SSDs recognize protein substrates and guide them into cavities inside the Clp hexamers, however the crystal structure of ClpY does not support this hypothesis [33]. In hexameric ClpY, the C-terminal domain of each monomer faces either the outside of an adjacent monomer or the solvent, therefore the C-terminal domain may not be involved in the transfer of protein substrates into the intra-hexamer cavity. Partial homology between *M. tuberculosis* ClpC1 and *E. coli* ClpB, ClpX, and ClpY suggests that the C-terminal domains of ClpC1 may assume conformations similar to analogous regions in ClpY (Figure 2). However, while the N-terminal region of ClpB is involved in interactions with protein substrates, the C-terminal region supports protein self-association. This result is consistent with the position of C-terminal domain in the crystal structure of ClpY [33]. Our results also indicate that contacts maintained by the C-terminal domain are necessary for stabilization of *M. tuberculosis* ClpC1 hexamer. The current study identifies a region within the C-terminal region of *M. tuberculosis* ClpC1 which primarily supports protein self-association (Figure 2).

In conclusion, our study demonstrates that the ATP-induced oligomerization is necessary for the chaperonic function of *M. tuberculosis* ClpC1. Also, a region at the C-terminus, between amino acids 720–747 in the small D2 domain, of *M. tuberculosis* ClpC1 is involved in the oligomerization of the protein and in turn in its function. However, the two N-terminal domains are not crucial for the ATPase and chaperonic activities of *M. tuberculosis* ClpC1.
